# My new home: study protocol for a prospective cohort study on the long-term personality development and short-term processes during transitions into nursing homes

**DOI:** 10.1186/s12877-025-06300-1

**Published:** 2025-08-19

**Authors:** Yang Sun, Cornelia Wrzus

**Affiliations:** Psychological Institute, Network Aging Research (NAR), Heidelberg University, Bergheimer Strasse 20, Heidelberg, 69115 Germany

**Keywords:** Later life relocation, Personality development, State, Social activity, Daily routine, Nursing homes

## Abstract

**Background:**

Personality is a crucial predictor of many life outcomes, including successful ageing. The proportion of nursing home residents is increasing annually among older populations. Relocations to nursing homes affect multiple aspects of older people’s lives, including their living environment, social interactions, and daily arrangements, which may indirectly affect their personality development. However, there is currently a lack of comprehensive assessments of how transitioning into a nursing home impacts personality development among older adults. This knowledge gap impedes our understanding of personality development in nursing home residents and also prevents our gaining insights into the related mechanisms and influences on personality development in later adulthood.

**Methods:**

“My New Home” will be a prospective cohort study designed to recruit 120 participants (age ≥ 60), including 60 older adults who will be relocating to a nursing home. This study will employ a series of longitudinal measurements, including a baseline measurement before relocation, daily measurements during the initial transition phase (for 28 days after moving), and monthly assessments during the adaptation phase (for 6 months). These measurements will comprehensively track the short-term state changes and long-term trait developments of personality before and after the older people’s relocations. Older adults’ personality traits will be assessed with the Big Five Inventory-2-Short. Additionally, this study will explore the moderating effects of older people’s age, gender, health status, cognitive function and subjective perceptions of living in nursing homes on the impact of relocation on personality traits. Furthermore, the study will test the hypothesis that the effects of relocations on personality traits are mediated through repeated influences on personality states, social activity, and daily routines.

**Discussion:**

We expect this study to provide a comprehensive and in-depth exploration of the mechanisms and processes by which relocations to nursing homes impact older people’s personalities. To our knowledge, this research will represent the first prospective cohort study that focuses on the personality development of nursing home residents. The research findings hopefully contribute to enhancing our understanding of the relationships between major life events and later life personality development.

## Introduction

As global fertility rates continue to decline and life expectancy increases, an increasing number of older adults will move to nursing homes in their final stages of life if no adult children can provide (sufficient) care. While some older individuals choose to reside in nursing homes for better care, a greater number are essentially forced to relocate to these facilities due to rapid deteriorations in their physical and cognitive functions [1], and maintaining and improving the well-being of residents in these institutions has become a significant societal concern [2, 3]. However, the adjustment to such new life environments also depends on people’s personality traits, and a major life event such as a later life relocation may lead to personality changes [4, 5]. Personality traits are commonly defined as relatively stable individual differences in emotion, behavior, and/or cognition [6], and are strongly associated with important life outcomes, including health, longevity, and successful aging [7, 8]. Extensive empirical findings have indicated that personality development and change occur throughout the lifespan, with life events playing a critical role in driving personality development [9–11]. Recent theories of personality development emphasize that major life events drive personality development in a bottom-up way through repetitive short-term processes like changes in state and daily behavior [12–14]. Despite this, the impact of moving to a nursing home on personality development in later life have not been examined. However, this major life event directly affects older people’s living environments, identities, and daily behaviors [1, 15, 16]. Overall, little is known about the process of personality development during transitions to nursing homes. This gap limits the evaluation and validation of theories on personality development in middle and old age and hinders a comprehensive assessment of the impacts of transitions to nursing homes. Considering this research gap, this study, using a series of specific measures related to personality development processes (both long-term and short-term), will thoroughly examine whether and how relocating to a nursing home in later life impacts personality development among older adults.

### The long-term development of personality during transitions to nursing homes

Examining the long-term mean changes in trait levels is key to investigating the impacts of life events on personality development [17]. Relocating to a nursing home is considered one of the most stressful life events an older adult can experience, and it affects almost every aspect of the older adult’s life, including daily routines, social engagements, and networks [18]. The relationship between this life event and personality traits is likely bidirectional, influencing and being influenced by personality traits both directly and indirectly. On one hand, according to the five-factor theory (FFT) [19], personality traits can influence older people’s decisions to move to nursing homes. This theory assumes that personality traits predispose individuals to experience specific life events and choose particular environments. For instance, previous research has indicated that higher agreeableness and lower conscientiousness are associated with a greater likelihood of using long-term care facilities [20].

On the other hand, changes in living environments, substantial reductions in social engagements, shrinking social networks, and increased life uncertainty after moving into nursing homes can all potentially serve as a pivot point for a person’s development. The paradoxical theory of personality coherence [21] suggests that individuals face unfamiliar situations, where past experiences do not aid in adapting to new environments. They may use environmental cues to guide and change their behavior, potentially leading to personality changes. As older adults transition from their previous living environments to nursing homes, their time previously used for existing social networks and daily routines significantly declines, and much of their time is spent interacting with caregivers and other residents. This change can introduce substantial environmental stress, requiring new residents to live in a more cooperative and accommodating manner, which might further increase their agreeableness, aligning with the “corresponsive principle” of personality development [22]. Similarly, based on this principle and the previous finding [20], it is reasonable to hypothesize that relocating to nursing homes will decrease older adults’ conscientiousness, as fewer daily (household) tasks must be accomplished. Additionally, extensive research on nursing home residents has indicated that during transitions to such facilities, individuals often exhibit lower emotional stability due to stressors experienced during relocations and in their new environments [23, 24]. Thus, we propose that relocation to nursing homes may lead to higher neuroticism in older adults. Overall, the current study will primarily investigate the first hypothesis (H1) that “relocation to nursing homes in later life leads to changes in Big Five personality traits, especially increases in agreeableness, decreases in conscientiousness, and increases in neuroticism.”

### Methodological and moderators in the study of relocations to nursing homes on personality development

Building on recommendations from previous researchers [25], this study will address four methodological issues to deepen our understanding of personality development during life events, especially during nursing home transitions, (a) accounting for the timing of the event (relocation) and charting the trajectory of trait changes; (b) considering pre-existing differences between individuals who do and do not relocate [26, 27]; (c) considering potential moderating factors that might influence the impact of the relocation on personality development to better assess the independent effect of the target event [11]; and (d) emphasizing the timing of “change,” exploring potential short-term processes in personality development [14, 28].

Specifically, we will utilize a prospective cohort study design, recording the relocation time and incorporating this into model testing. Older people’s personality traits will be measured on multiple occasions (e.g., before relocation, and during transition and adjustment phases) to capture the trajectory of trait changes. Additionally, by establishing a control group of people remaining in private residences, we might better distinguish the impacts of moving to nursing homes on traits from the general development patterns of personality in this age group. Some previous research indicated that individuals often respond differently to the same life transitions [29, 30]. Individual differences might be key to explaining the variable impacts of the same life events on personality traits [25, 31]; considering and quantifying potential moderating variables is one method to address this developmental heterogeneity. We hope to extend previous research findings by including and hypothesizing that a resident’s age, gender, health status, cognitive function, and subjective perception of residing in nursing homes moderate the impact of relocations on older adults’ personality traits (H2).

#### Age

Typically, residents in nursing homes are over 65 years old, but in reality, their ages span a broad range, with some residents aged over 100 years, showing significant age variability [32]. Even among older age groups, the personality development trajectories of young-old adults (65–85 years) and oldest-old adults (85 + years) differ [33, 34]. Comparatively, younger older adults have stronger psychosocial reserves, showing higher personality plasticity and better adjustment to unfamiliar environmental impacts, whereas oldest-old adults are more directly impacted by unfamiliar environments due to widespread age-related functional limitations that limit their ability to adjust and cope with new changes, potentially weakening the driving effect of relocation to nursing homes on their personality development.

#### Gender

We will also hypothesize that relocating to nursing homes might more significantly impact the personality development of older female adults. In previous research on environmental adaptation and psychological adjustment of nursing home residents, female residents often exhibited higher levels of psychological adjustment and environment adaptability [35], and this cumulative adjustment process aimed at adapting to the new environment might further promote the personality development of female residents [36, 37].

#### Health status

Extensive research has indicated that the deterioration of health status in later life is associated with a broad decline in the Big Five personality traits (including extraversion, agreeableness, conscientiousness, openness, and emotional stability) (e.g [34, 38, 39]).,. Older adults often move into nursing homes due to health issues and functional limitations, and their health status may potentially influence the link between relocation and personality development.

#### Cognitive function

Decline in cognitive function is a common factor contributing to nursing home admission, as well as a key predictor of how well individuals adjust after relocation [40]. Empirical studies have shown that impaired cognitive function reduces older adults’ ability to maintain their established lifestyles and adapt to new environments. It is also significantly associated with declines in personality traits that reflect psychological maturity, such as emotional stability and conscientiousness [38, 41]. We hypothesize that older adults with relatively intact cognitive function will adapt more easily to the transition into a nursing home, and that any negative impact on their personality traits—particularly emotional stability— will be less pronounced.

#### Subjective perceptions of residing in nursing homes

Recent research has highlighted the importance of measuring individuals’ subjective perceptions of events, as different perceptions of the same event can partly explain the different impacts of the same event on personality development (reviews in [31, 42]). Examining the moderating effect of older adults’ subjective perceptions of residing in nursing homes in this study should allow for a more comprehensive and systematic exploration of the mechanisms by which relocations impact personality development in later life.

Finally, considering that the repeated momentary changes in trait-related states, daily routines, and social activity after the event (relocation) might be key factors in the long-term development of older adults’ personalities [14, 43], we will record and examine the short-term changes in states, daily routines, and social activity during the initial phase of transition to nursing homes (within 28 days after relocation) and explore their associations with long-term personality development. Next, we detail the necessity and feasibility of examining the aforementioned short-term changes.

### Short-term processes of personality change during relocation transitions: States, daily routines, and social activity

Life events may impact personalities in ways both direct and immediate, often reflected swiftly in changes in personality states and alterations in behavior, emotions, and cognition following events [44, 45]. However, isolated life events alone are insufficient to induce personality changes; the actual catalysts for trait modification are the changes in social roles, interactions, and daily routines triggered by these events [26]. To comprehensively understand the process of personality change caused by life events, it is crucial to consider the role of transient states and immediate changes that result from events. Consequently, this study will assess the personality states, social activity, and daily routines of older adults after moving into nursing homes to explore whether and how relocation influences long-term personality development through the associated repeated short-term processes.

Personality states refer to the transient patterns of behavior, emotions, and cognition an individual exhibits at specific times and in specific situations, considered to be momentary expressions of personality traits [46]. Benefiting from the widespread application of experience sampling methodology (EMA) in personality research, researchers have been able to more accurately describe the relationship between personality states and traits [46–48]. Earlier frameworks like the sociogenomic model of personality [49], the framework for self-regulated personality development [48], as well as the more recent TESSERA (triggering situations, expectancies, states and state expressions, and reactions) framework [14, 50], emphasize that the recurrent short-term states following exposure to trait-relevant situations are a prerequisite for long-term personality development. According to the TESSERA framework, the relocation to a nursing home in later life disrupts the familiar life routines and social environments of the new residents, potentially acting as a triggering situation for changes in personality traits. Observing the immediate changes in personality states (e.g., social activity and daily routines) post-relocation and their interplay could provide valuable insights into the underlying mechanisms of personality trait development during transitions to nursing homes. Notably, a change in one state does not only lead to a change in the corresponding trait [51]. According to network theory [52], a change in one central state, due to its centrality in the individual’s personality network, will not only directly affect its corresponding traits but may also affect the stability and change of states in other domains through the network’s connectivity pathways. For example, increased agreeableness might be achieved by increasing social interaction and interpersonal trust, which in turn enhance extraversion, or by reducing neuroticism manifested from social anxiety. Such changes in state can form a chain reaction throughout personality development, evolving over time and exhibiting different characteristics at different life stages or under various environmental factors. Thus, we will further hypothesize that relocating to nursing homes triggers changes in the Big Five personality states of older adults, and that the interactions among these states will collectively contribute to the long-term development of personality traits across domains.

Personality is closely linked to social activity, with the structure of the Big Five personality traits reflecting reliable correlations, particularly with agreeableness and extraversion in social contexts [53]. From the perspective of the personality-relationships transaction [54], the association between life events and personality change can be seen as a result of the interaction between social activity and personality. Research has shown that a later life relocation, resulting in decreased social activity, correlates with lower extraversion and increased neuroticism among residents [55]. Moreover, according to the personality and social relationships framework (PERSOC) [56], changes in state-level personality and social activity, and their interactions, provide mechanisms for the long-term development of personality traits.

Disruptions of daily routines and a reduction in the diversity of daily life are among the most direct impacts of relocations to nursing homes for older adults [3, 57]. In nursing home environments, many routines, like dining and social activities, are strictly managed externally. The uniform routine practices of nursing homes often do not match the previous lifestyle habits of older people, making it difficult for them to maintain their original daily routines. Additionally, health status, facility conditions, and standardized schedules limit the diversity of daily routines available for the older adults. An individual’s daily routine is often a specific reflection of their personality, and daily routines may provide opportunities for or impose restrictions on the expression of certain states [58]. Disruptions in daily routines and reductions in routine diversity can subtly influence personality traits. These cumulative subtle changes may be an important process by which life events, such as relocating to a nursing home, change personality traits through a bottom-up mechanism.

Overall, we will hypothesize that during the transition to nursing homes, the changes in personality states, social activity, and daily routines and their interactions constitute short-term processes of trait development (H3). These repetitive short-term processes subsequently influence the long-term development of personality traits.

### Summary

**Fig. 1:**
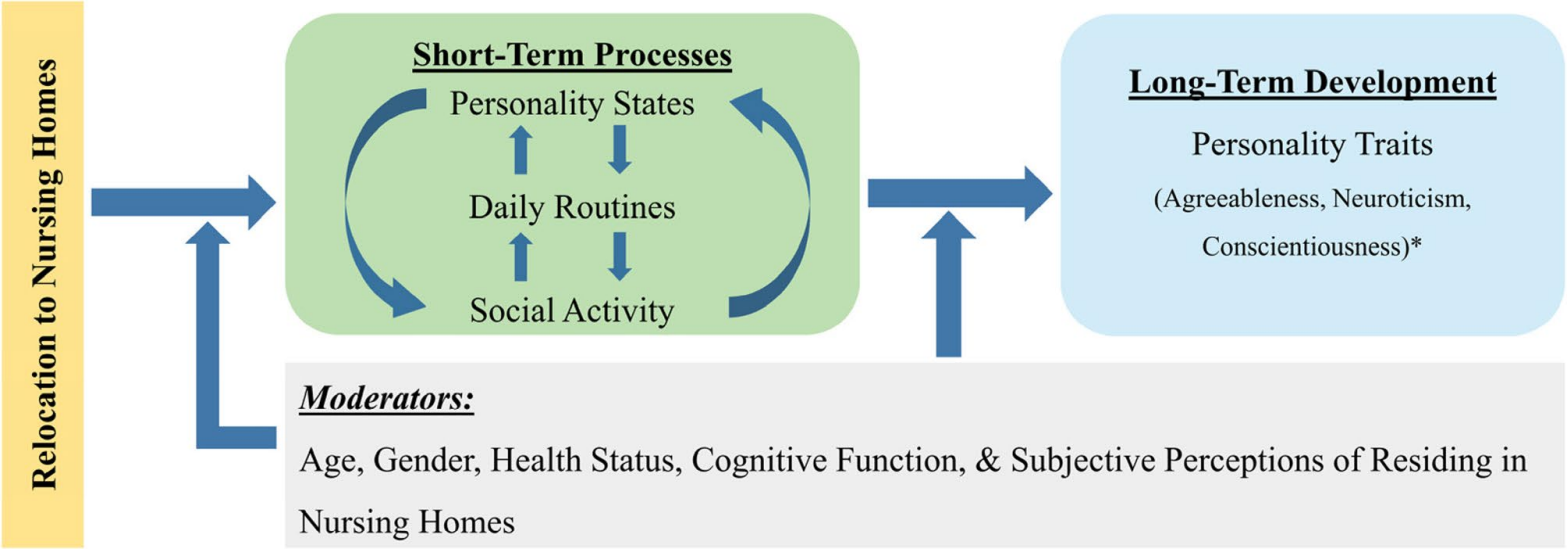
Research Framework

With this study we hope to conduct a comprehensive and in-depth longitudinal investigation of the long-term development and short-term processes of personality change during transitions to nursing homes in old age, as illustrated in our research framework (Fig. 1). We hope to provide new and reliable insights regarding personality development in old age and guide further personality interventions to enhance the well-being of nursing home residents. In summary, this study will explore the following questions and hypotheses:

#### Question 1

Does relocation to a nursing home affect the personality traits of older adults?

- H1. Relocation to nursing homes will significantly impact the personality traits of older adults, resulting in higher agreeableness and neuroticism, and lower conscientiousness.

#### Question 2

How do age, gender, health status, cognitive function, and subjective perceptions about the relocation moderate these effects?

- H2. The impact of relocation on personality traits will be moderated by age, gender, health status, cognitive function, and subjective perceptions of the relocation.

#### Question 3

Through what mechanisms does relocation to nursing homes affect personality traits?

- H3. The long-term development of personality traits will be mediated by changes in personality states, social activity, and daily routines post-relocation.

## Methods

### Participants

This study plans to recruit 120 participants aged 60 years and above. The sample size estimation is based on previous related studies and determined through a power analysis using G*Power (version 3.1.9.7). It is anticipated that including 109 participants in this study will achieve 80% power at the 0.05 α level, with an expected medium effect size (f² = 0.15). 10%, that is, 11 additional participants will be included to account for potential attrition and ensure robust statistical analysis. The participants will be recruited from two groups: a relocation group (experimental group), consisting of 60 older adults who will move into a nursing home, and a control group, consisting of 60 older people who have not relocated in the past 2 years and continue to reside in their private homes.

#### Recruitment

To ensure the representativeness of the sample and minimize selection bias, this study will use a purposive sampling strategy to recruit older people including those of different ages, genders, and health statuses. Recruitment will be conducted through multiple concurrent channels, aiming to maximize the diversity and representativeness of the sample, covering different socio-economic backgrounds and residential situations. Major recruitment channels include online advertisements, email lists, flyers, news articles, and acquaintance referrals. These channels will be used for recruiting participants for both the experimental and the control group. Additionally, considering that most nursing home residents are either directly transferred from hospitals or registered on waiting lists before admission to nursing homes, this study will also collaborate with local hospitals and nursing homes to send recruitment information to potential participants. This study is currently in the initial phase of collaborator recruitment, and participant recruitment has not yet begun.

#### Eligibility criteria

After providing informed consent, all potential participants will be screened for eligibility. Inclusion criteria are being at least 60 years old and having sufficient cognitive functioning to independently complete all study assessments. Cognitive functioning will be evaluated in two stages. First, individuals with a medically confirmed diagnosis of severe cognitive impairment will be excluded at recruitment. Second, all participants will complete the Montreal Cognitive Assessment (MoCA) during the baseline assessment. The MoCA is a widely used instrument for evaluating overall cognitive functioning in older adults. A score below 18 will be considered indicative of moderate to severe cognitive impairment and insufficient capacity to complete the intensive follow-up survey [59]. These individuals will not proceed to the follow-up phase of the study. To reduce potential confounding effects related to recent residential changes, participants in the control group must not have relocated within the past two years.

### Design

**Fig. 2:**
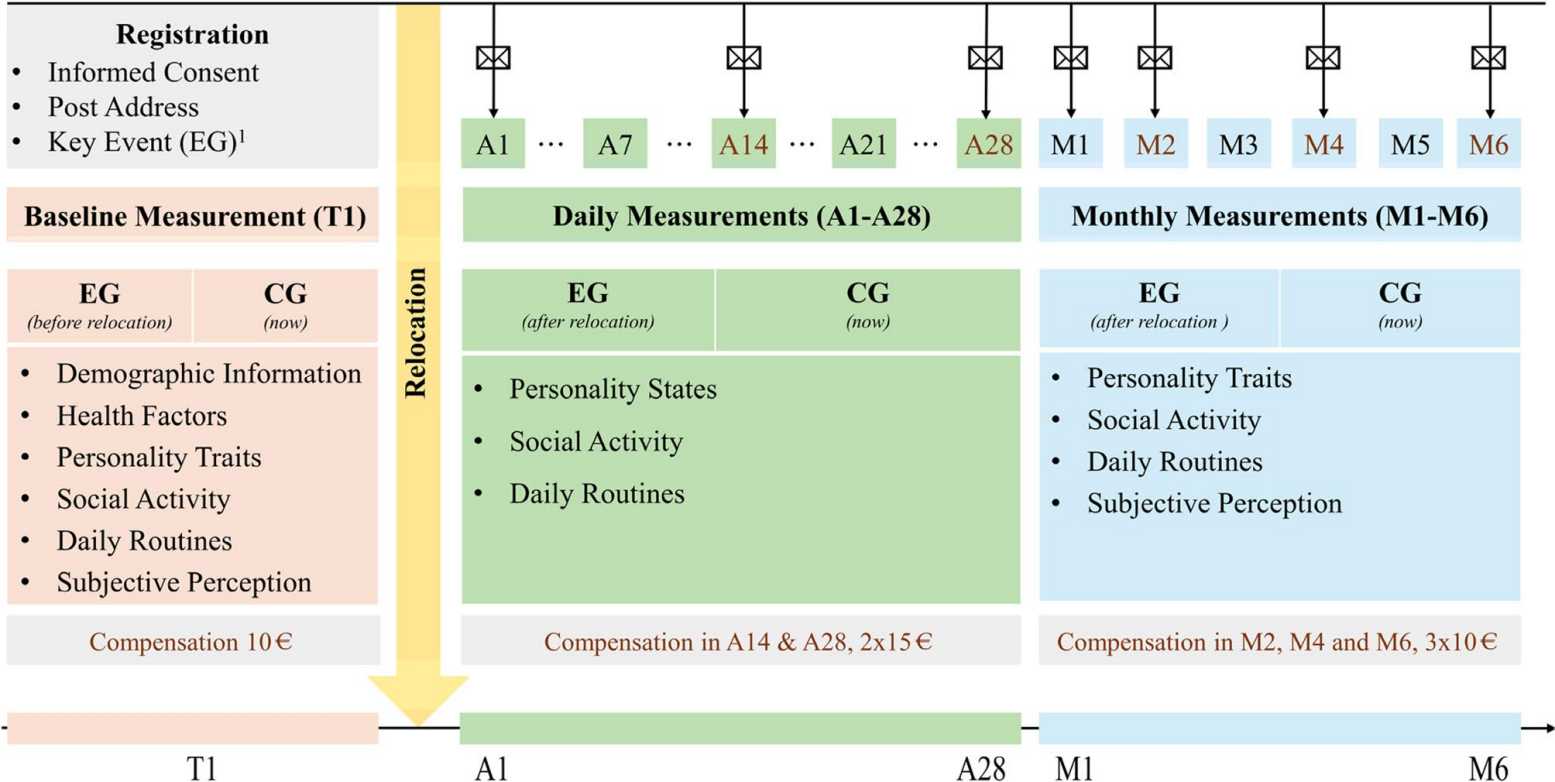
Research Flowchart. Note EG = experimental group; CG = control group. ^1^Record the possible time of relocation and organize how and when the questionnaires will be distributed for daily measurements. Reminders will be sent to participants who need them on the date the envelope logo appears to ensure that participants are able to successfully complete the study

This study will use a prospective cohort design with three main phases (see Fig. 2), including a baseline assessment, a daily diary assessment for 28 days, and a monthly assessment for 6 months. For older adults relocating to nursing homes (i.e., experimental group), these phases correspond to the transitional stages of entering a care facility: preparation, initial transition, and adaptation. Each phase will include specific measurement points to capture the psychological and social dynamics associated with transitioning into a care facility. All data collection will be carried out by trained researchers to ensure data consistency and accuracy. The study protocol has been reviewed and approved by the Ethics Committee of the Institute of Psychology at Heidelberg University (Approval No: AZ Sun 2023 1/2).

### Procedures

Before the commencement of the study, all eligible respondents will be informed about the general nature and objectives of the research. After providing informed consent, participants will undergo a series of three assessment phases over a period of 7 months. When participants choose to withdraw or are unable to complete the study, the specific reasons (e.g., health, interest, study burden) and timing of the withdrawal will be recorded and saved, and factors that may affect the results will be adjusted in the final report. Additionally, measures such as regular follow-ups and reminders will be implemented to minimize participant dropout.

If a control-group participant relocates to a nursing home within one month following the baseline assessment, they will be invited to continue in the study, with renewed consent, and will restart the daily assessment phase (Phase 2). If a sufficient number of such cases is reached (*n* ≥ 10), these participants will be exploratorily reclassified into a separate subgroup (“unexpected relocation subgroup”) to allow for the examination of personality change trajectories following unanticipated residential transitions. If the number of such cases remains small (*n* < 10), these participants will be recorded as dropouts and will not be analyzed as a distinct subgroup.

If a relocation occurs after completion of the daily assessment phase (i.e., after completing the first two phases of measurement), monthly assessments (Phase 3) for those participants will be discontinued. The timing and reason for relocation will be recorded, but these individuals will not be reassigned to the relocation subgroup to avoid biases occurring from repeated measurements. To maintain the intended sample size, new participants will be recruited to replace those who withdraw.

#### Preparation phase (Baseline measurement, T1)

Baseline assessments will be conducted for experimental-group participants before relocation, at a mutually agreed-upon time, and for control-group participants immediately following enrollment. Measures collected at baseline include demographic characteristics, self-rated health, cognitive function, Big Five personality traits, social activity, daily routines, and subjective perceptions of nursing home living. Trained research assistants will administer all baseline assessments and subsequently provide participants with instructions about the procedures for daily and monthly follow-up assessments, including questionnaire distribution and collection.

#### Initial phase of transition (Daily surveys, A1-A28)

Beginning on the day of relocation, participants will complete daily surveys for 28 days to assess their personality states, social activity, and daily routines. The control group will start their daily surveys 1 month after the baseline measurement and receive the same measurements.

#### Adaptation phase (Monthly surveys, M1-M6)

Monthly follow-up surveys will begin 1-month post-relocation and continue for 6 months for the experimental group. This phase will continue to track changes in older adults’ personality traits with the administration of measures consistent with the baseline measurements, but without repeated measures, for relatively stable demographic information. The control group will commence monthly surveys 2 months post-baseline measurement, aligning the timeline and measurements with the experimental group to evaluate stability or natural change in their condition over time (see Fig. 2 for details).

### Measures

#### Accommodation type

The type of accommodation in which the older people are residing will be recorded as a dichotomous variable (nursing home = 0; private home = 1).

#### Personality traits

The Big Five Inventory-2-Short (BFI-2-S) will be used to assess the five personality traits of agreeableness, conscientiousness, extraversion, neuroticism, and openness to experience in older adults. The BFI-2-S is simplified from the 60-item BFI-2 inventory and consists of 30 items, six items per domain, rated on a 5-point Likert scale from 1 (strongly disagree) to 5 (strongly agree). The BFI-2-S has been validated for strong internal consistency and convergent validity, ensuring that the key dimensions of personality are captured effectively while reducing the burden on respondent [8].

#### Personality states

In this study, we will employ the adjective measurement approach proposed in [60] for bipolar disorder, using a 10-item bipolar rating scale to assess participants’ daily personality states. Each personality dimension will be assessed through two items, using a 1 to 7 Likert bipolar rating scale, contrasting two adjectives: *extraversion* (quiet, assertive vs. talkative, assertive); *agreeableness* (cold, resentful vs. empathetic, forgiving); *conscientiousness* (chaotic, lazy vs. organized, hardworking); *neuroticism* (pessimistic, mood-optimistic vs. emotionally stable); and *openness* (disinterested, uncreative vs. interested, creative). Except for the two items in the neuroticism dimension, which use reverse scoring, the scoring for all other dimensions will be positively oriented.

#### Social activity

Social activity will be assessed by quantifying the frequency, manner, and quality of interactions with key social relationships, including partners, children, grandchildren, other relatives, friends, caregivers, and neighbors. Interaction frequency will be quantified based on the regularity of contact: multiple times a day (6 points), once a day (5 points), several times a week (4 points), once a week (3 points), two to three times a month (2 points), and once a month (1 point). The manner of communication will be coded based on the intimacy and immediacy of the communication: face-to-face (3 points), telephone (2 points), and video communication (1 point). The quality of interactions and anticipation of future interactions will each be assessed on a 5-point scale, ranging from “very unpleasant or not at all looking forward to” (1 point) to “very pleasant or very much looking forward to” (5 points). Each participant’s total social activity score will be calculated by summing the scores for interaction frequency, manner, quality, and expectations across all types of social contacts.

#### Daily routine

This study will also assess the daily routine of older adults, using a customized questionnaire called the Scale of Older Adults’ Activities Routine (SOAR) [61] and the Activities of Daily Living (ADL) types [62]. The questionnaire categorizes routines into 16 types: self-care, cooking, housekeeping, working, shopping, personal relaxation, group activities, volunteering, social visits, receiving visitors, outdoor sports, indoor exercise, prayer, recreational activities, communication, and travel. Two measurement methods are utilized: monthly and daily questionnaires. On the monthly questionnaire, participants rate each routine based on frequency, with scores ranging from 1 (several times a day) to 8 (never). On the daily questionnaire, the scoring ranges from 1 (seven times a day) to 8 (never). The overall routine frequency for an individual is calculated by averaging the frequency scores of all routines. Additionally, routine diversity is assessed by counting the number of routine categories in which participants report engagement, excluding those rated as “never.”

#### Subjective perceptions of residing in nursing homes

This study will also employ two items selected from the Event Characteristics Questionnaire (ECQ) [42], specifically from the dimensions of valence and challenge, to assess the subjective perceptions of older people residing in nursing homes. These items were suitably adjusted to better align with the objectives and design requirements of our research. Specifically, the assessment items will include “Residing in a nursing home would be positive for me” and “Residing in a nursing home would be stressful for me.” These items will be scored using a 5-point Likert scale, ranging from 1 (does not apply at all) to 5 (applies completely).

#### Cognitive function

Participants’ cognitive functioning will be assessed using the Montreal Cognitive Assessment (MoCA) [63], a well-validated instrument designed to evaluate overall cognitive functioning in older adults. The MoCA assesses multiple cognitive domains, including visuospatial and executive abilities, memory (recall and registration), attention, language, abstraction, and orientation. It will be administered by trained research staff using standardized procedures. The total score ranges from 0 to 30, with higher scores indicating better cognitive functioning. According to revised scoring criteria, a total score below 18 is considered indicative of moderate to severe cognitive impairment in this study [59].

#### Socio-demographic and health variables

This study will assess the socio-demographic variables of chronological age and gender (1 = male, 2 = female). Participants’ self-perceived health status will be assessed using three items, including “How would you rate your current state of health?” with responses ranging from 1 (excellent) to 5 (poor); “How severe is your pain or physical discomfort at the moment?” with responses ranging from 1 (not present) to 5 (extreme); and “Do you currently suffer from a chronic illness?” with responses coded as 1 (no) and 2 (yes).

### Data analysis plan

The data analysis for this study will primarily be conducted in R (Version 4.2.3; The R Foundation for Statistical Computing, Vienna, Austria). To test hypotheses 1 and 2, we will analyze the effects of relocations on personality traits using our baseline and monthly measurements data and examine the moderating effects of age, gender, health status, and subjective perceptions of residing in nursing homes. This analysis will be conducted through a series of multilevel models that incorporate robust sensitivity analyses to account for any potential biases due to participant dropout or transition from control to experimental groups.

Moreover, multivariate time-series analyses will be employed to explore the dynamic relationships between personality state, social activity, and daily routine from the daily measurements. Upon completion of these analyses, a series of structural equation models (SEMs) will be constructed using the baseline, daily, and monthly data to examine the mediating roles of personality state, social activity, and daily routine in the relationship between relocation and changes in personality traits, possibly validating H3.

## Discussion

The “My New Home” study outlined in this research protocol will hopefully offer a substantial advancement concerning later life personality development and the effects of relocations to nursing homes in later life. Due to high attrition rates and tracking difficulties, most traditional longitudinal panel studies have excluded nursing home residents (e.g., SOEP, MIDUS, HILDA), resulting in a persistent lack of understanding about the processes and influencing factors of personality development in this demographic [64, 65]. To our knowledge, the present future study will be the first longitudinal study to focus on the personality development of nursing home residents. We confidently anticipate that the findings from this study will provide reliable new insights regarding the importance of later life relocations as catalysts of personality development.

Our study protocol will have several strengths. First, this will be a comprehensive prospective cohort study design, and we have developed a corresponding measurement plan based on the transition phases into nursing homes, including baseline assessments (preparation phase before moving), daily assessments (initial transition phase), and monthly assessments (adaptation phase). This approach will meticulously track the personality development trajectories of residents at different stages of transition to nursing homes, hopefully overcoming the limitations of previous studies that relied solely on pre- and post-measurements. Moreover, unlike traditional annual follow-up studies [10, 66], our approach should be able to more accurately capture the transient responses of personalities, thereby gaining a deeper understanding of the short-term changes and long-term developmental mechanisms of personality development (e.g [14, 67]).,. The results might capture the dynamic trajectories of personality development in adulthood triggered by a move to a nursing home, providing reliable evidence for the causal relationship between major life events and personality in late adulthood. Second, by setting up a control group, this study should be able to effectively distinguish between the effects of relocating to nursing homes on personality development from the general pattern of personality change in late adulthood. Third, our research methodology will also consider individual differences, and including relevant moderating variables and covariates in the model will allow for a comprehensive and in-depth exploration of the impact of later life relocations on personality traits, also possibly contributing to the validation and support of previous research findings. Finally, by employing techniques like time series and SEM, we will be able to obtain reliable statistical models of the dynamic trajectory of personalities among older people before and after relocation.

One of the main challenges to the implementation of this study will be the difficulty of recruiting a sufficient number of older people who intend on moving to a nursing home in a single city or region. Typically, due to limitations of available resources, nursing homes often have long waiting lists, and homes often only accept a few new residents each year; for example, in the Heidelberg region, some nursing homes may only welcome 1–2 new residents per year. A possible solution to this dilemma is to expand the scope of the research and recruitment through cross-regional/country collaboration with more researchers. Such an approach might not only contribute to a smoother implementation of this study but might also enhance the robustness and generalizability of its findings.

As second challenge might be related to sample composition. Systematic differences between people living in nursing homes and in private homes, which affect both the initial participation in the study (i.e., selection biases) and the selective drop-out due to health issues (i.e., survivorship bias). Moreover, because participants with moderate-to-severe cognitive impairment (MoCA < 18) were excluded, the nursing-home cohort under-represents the most cognitively frail residents, potentially attenuating relocation effects and limiting generalizability to the full spectrum of institutionalized older adults. We will handle these biases through (a) thorough description of both groups with respect to demographic, socioeconomic, and health indicators, and (b) attrition analyses over time.

Overall, the proposed “My New Home” research, via its comprehensive longitudinal study design, will aim to provide new and practical insights into the impact of later life relocations on personality development. We hope our future findings both help guide future interventions aimed at improving nursing home residents’ well-being and promote successful aging.

## Data Availability

No datasets were generated or analysed during the current study.

## Abbreviations

TESSERA: Triggering situations, expectancies, states and state expressions, and reactions
EG: Experimental group
CG: Control group
SEM: Structural equation modelling
SOEP: Socio-economic panel
MIDUS: Midlife in the United States
HILDA: Household, income, and labor dynamics in Australia
